# *STAT3* and *TP53* mutations associate with poor prognosis in anaplastic large cell lymphoma

**DOI:** 10.1038/s41375-020-01093-1

**Published:** 2020-11-27

**Authors:** Cosimo Lobello, Boris Tichy, Vojtech Bystry, Lenka Radova, Daniel Filip, Marek Mraz, Ivonne-Aidee Montes-Mojarro, Nina Prokoph, Hugo Larose, Huan-Chang Liang, Geeta G. Sharma, Luca Mologni, David Belada, Katerina Kamaradova, Falko Fend, Carlo Gambacorti-Passerini, Olaf Merkel, Suzanne D. Turner, Andrea Janikova, Sarka Pospisilova

**Affiliations:** 1 Center of Molecular Medicine, Central European Institute of Technology (CEITEC), Masaryk University, Brno, Czech Republic; 2 Department of Internal Medicine—Hematology and Oncology, University Hospital Brno and Medical Faculty MU, Brno, Czech Republic; 3 Institute of Pathology and Neuropathology and Comprehensive Cancer Center Tübingen, Eberhard Karls University, Tübingen, Germany; 4 Division of Cellular and Molecular Pathology, Department of Pathology, University of Cambridge, Cambridge, UK; 5 Department of Pathology, Medical University of Vienna, Vienna, Austria; 6 Department of Medicine and Surgery, University of Milano-Bicocca, Monza, Italy; 7 4th Department of Internal Medicine—Hematology, Charles University Hospital and Faculty of Medicine, Hradec Králové, Czech Republic; 8 Fingerland Department of Pathology, Charles University Hospital and Faculty of Medicine, Hradec Králové, Czech Republic

**Keywords:** Genetics research, Cancer genomics, Cancer genomics

## To the Editor:

Systemic anaplastic large cell lymphoma (sALCL) encompasses two distinct clinical entities of T-cell non-Hodgkin lymphoma: anaplastic lymphoma kinase-positive (ALK+) ALCL and ALK-negative (ALK−) ALCL. These entities are characterized by either the presence or absence of an ALK translocation. It has been reported that ALK+ ALCL has a better prognosis compared to ALK−, with a 5-year overall survival (OS) of 70–80% versus 40–60%, respectively, [1–3]. Furthermore, more than 30% of ALK+ ALCL patients relapse [4, 5]. Despite the distinction between the two sALCL subtypes, frontline treatment for adults is similar and is based on CHOP or CHOEP, instead pediatric ALCL patients are mainly treated following the ALCL99 protocol [6–8]. Whilst high-throughput genomic studies in sALCL have shown recurrent genetic alterations, their association with outcome has not been fully investigated [9–13].

In this study, the mutational landscape of sALCL patient tumors was investigated to discover potential biomarkers that may improve risk stratification and patient management.

A cohort of 82 sALCL patient tumors (47 ALK+ and 35 ALK−) and 6 ALCL cell lines (4 ALK+, 2 ALK−) (Table S1) were subjected to deep targeted next-generation sequencing analyzing the whole coding regions of 275 cancer related genes (Table S2). The average depth achieved across all the samples sequenced was ~2000×. Sequencing data are available at Sequence Read Archive (https://www.ncbi.nlm.nih.gov/sra/, SRA identifier PRJNA602225).

**Fig. 1:**
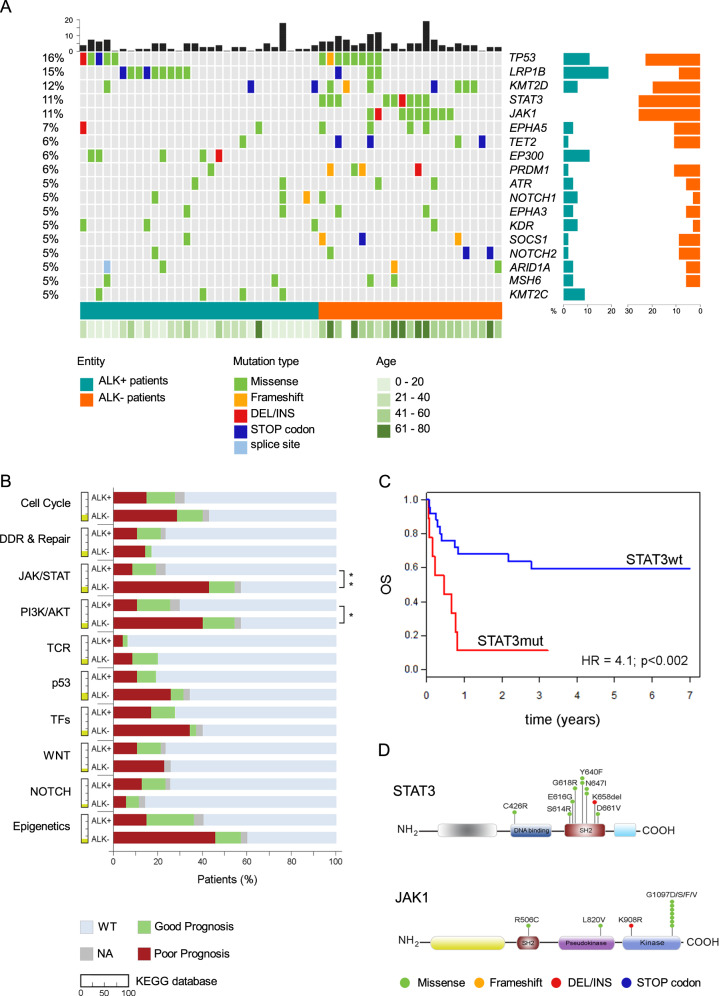
**Mutational landscape in sALCL reveals prognostic biomarkers**. **a** Oncoplot shows the genes mutated in at least 5% of the entire cohort. The percentage is shown on the left axis. Each column represents a patient, ALK+ in dark green and ALK− ALCL patients in dark orange. The black bars on the top represent the number of mutated genes in each patient. On the right axis, the frequency of mutated gene in ALK+ (dark green) and ALK− (dark orange) ALCL patients. The green bar on the bottom shows the age of each patient. Mutation types are represented in different colors as shown in the legend. **b** Percentage of patients harboring at least one mutated gene in ten biological pathways. The yellow colored portion next to each pathway indicates the percentage of genes present in our panel that belong to that specific pathway according to the KEGG database. For each pathway shown, the patients are divided according to prognosis; red: patients with poor prognosis; green: patients with good prognosis; gray: patients for whom clinical information is not available (NA); light blue patients wildtype (WT) that do not harbor mutated genes in that specific pathway. The patients are represented as percentage of the total. DDR and repair DNA damage response and repair pathway, TFs transcription factors. Fisher’s exact test: *p < 0.05, **p < 0.01 and ***p < 0.001. **c** 7-year OS of ALK− ALCL patients according to *STAT3* status: *STAT3* mutated (red) versus *STAT3* wt (blue). *P* values and hazard ratios (HR) shown were determined by the Cox proportional hazards model. **d** Schematic representation of STAT3 and JAK1 domains and the position of the variants.

Male subjects were predominant in both subgroups of our cohort, 57.4% in ALK+ versus 67.6% in ALK−. ALK+ patients were significantly younger than ALK− patients with an average age of 22.7 (3–61) and 55.2 (27–81) years, respectively. ALK+ ALCL patients had a longer survival than ALK− ALCL with a 7-year OS of 77.6% and 46.7%, respectively, and with 7-year progression free survival (PFS) being comparable at 58.7% for ALK+ and 44.1% for ALK− patients (Fig. S1). The first line of treatment for all the adult patients was systemic chemotherapy, and most of the childhood ALK+ ALCL patients (80%) were treated following the ALCL99 or ALCL98 protocols. Although ALK+ patients have a longer OS, more than 30% relapsed after first-line treatment. Among the 275 genes analyzed, we identified 148 (54%) genes harboring at least one mutation throughout the entire cohort; 132 genes among the patients and 43 among the cell lines, with 27 genes in common (Fig. S2, Table S3). Overall, 72 out of 82 (88%) patients carried at least one mutation within the genes analyzed. We detected an average of 4.2 mutations per patient in ALK− ALCL and an average of 2.7 in ALK+ ALCL. The most recurrently mutated gene in the entire cohort was *TP53* found in 16% of sALCL patients (11% ALK+, 23% ALK− and in all ALK+ cell lines). Interestingly, for the ALK+ group, mutated *TP53* was more frequent in young patients (*p* < 0.04). *LRP1B* was prevalently mutated in ALK+ patients (19%) and in three cell lines. *STAT3* and *JAK1* were mutated solely in ALK− ALCL, both with a prevalence of 26%, and were the most mutated genes in this group (Fig. 1a). Recurrent mutations were detected in epigenetic modifier genes also recently reported to be frequently mutated in BIA-ALCL [14]. *KMT2D* and *TET2* were found mutated in ALCL patients regardless of ALK status and *EP300* and *KMT2C* only in ALK+ patients. Pathway enrichment analysis showed a significant enrichment in mutated genes involved in JAK/STAT (*p* < 0.003) and PI3K/AKT signaling pathways (*p* < 0.02) for ALK− ALCL compared with ALK+ ALCL (Fig. 1b). We investigated possible correlations between the existence of mutations in the most mutated genes and the clinical characteristics of our cohort. Poor prognostic outcome was defined as patients meeting at least one of the following criteria: deceased, unresponsive to treatment and/or disease relapse. The most recurrently mutated genes in the poor prognostic sub-cohort independent of ALK status were *TP53* (27%), *STAT3* (24%), *EPHA5* (16%), *JAK1* (16%), *PRDM1* (13.5%), *LRP1B* (11%) and *KMT2D* (11%). Considering only refractory/relapsed ALCL patients, mutations within *TP53* (28%) and *EPHA5* (19%) were the most common (Table S4). In relation to the prognosis, ALK+ patients did not show any significant difference in the signaling pathways affected by mutations. On the contrary, the JAK/STAT (*p* < 0.005) and PI3K/AKT pathways (*p* < 0.036) were enriched in ALK− ALCL patients with an inferior outcome (Fig. 1b). Pathogenetic variants of *STAT3* were detected in 9/35 (26%) of ALK− ALCL patients. Mutations were located mainly within the SH2 domain (S614R, E616G, Y640F, N647I, K658delinsNM and D661V) and in one case within the DNA binding domain (C426R). Mutated *JAK1* was detected in 9/35 (26%) of ALK− ALCL patients and of those, 6/9 were at the hotspot codon 1097 (G1097D/F/N/S) (Fig. 1d). For four patients, *JAK1* was mutated together with *STAT3*, thereby emphasizing the importance of the JAK/STAT signaling axis. To evaluate the prognostic value of mutations in the JAK/STAT pathway, we performed Cox regression analysis and showed that ALK− ALCL patients harboring *STAT3* and/or *JAK1* mutation have a shorter OS (hazard ratio [HR] = 2.8; 95% confidence interval [CI], 1.1–7.1, *p* < 0.03) (Fig. S3A). Furthermore, the prognostic value of the most mutated genes in ALK− ALCL: *STAT3* (9/35), *JAK1* (9/35), *TP53* (8/35) and *KMT2D* (7/35) were investigated. Cox regression analysis showed that patients with *STAT3* mutations have a significantly shorter OS compared to those with wild-type *STAT3* (HR = 4.1; 95% CI, 1.56–10.71, *p* < 0.002) (Fig. 1c). In addition, while *JAK1* and *KMT2D* mutations did not significantly correlate with OS (*p* < 0.2 and *p* < 0.3, respectively), *TP53* mutations clearly displayed the correlation (*p* < 0.01) (Fig. S3B–D). To further confirm that mutations in *STAT3* are associated with shorter OS, we applied Akaike’s informative criteria model to the four aforementioned genes. *STAT3* mutations were found to be the best predictor of OS in ALK− ALCL (Table S5). Moreover, no significant differences were found between mutation status of these genes with age, gender, disease stage, eastern cooperative oncology group performance status or age-adjusted international prognostic index (AA-IPI). As expected [9, 13, 15], expression of p-STAT3 (Y705) was detected at a high level in all ALK− ALCL patients harboring *STAT3* mutations, although low/medium expression of p-STAT3 was also detected in *STAT3* wild-type patient tumors (Fig. S4, Table S6). Mutations in the *LRP1B* gene were detected in 12/82 (15%) of sALCL patients (19% ALK+ and 9% ALK−) and three cell lines. Since *LRP1B* was the most recurrently altered gene in ALK+ ALCL, we assessed its possible association with outcome, but no differences were found between mutated and nonmutated patients.

**Fig. 2:**
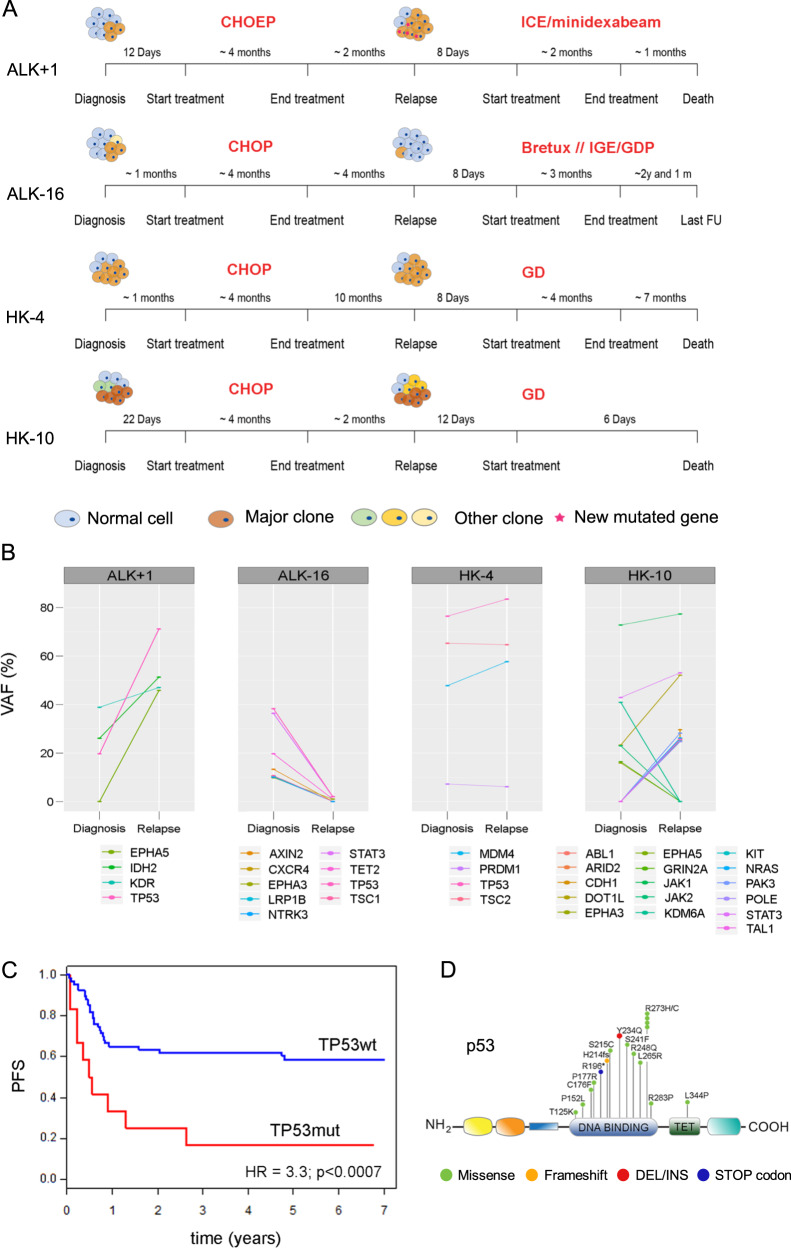
**Diagnosis versus relapse showed mutated*TP53*to be associated with a shorter PFS in sALCL**. **a** Schematic representation of four patients sequenced at diagnosis and at relapse highlighting their clinical path and treatment. Meaning of the color is described in the legend. CHOEP chemotherapy with cyclophosphamide, doxorubicin, etoposide, vincristine and prednisone, CHOP chemotherapy with cyclophosphamide, doxorubicin, vincristine and prednisone, ICE chemotherapy combination that includes ifosfamide, carboplatin and etoposide, bretux: brentuximab vedotin, GDP gemcitabine, dexamethasone, and cisplatin, GD gemcitabine and docetaxel, FU follow up. **b** Changes in mutational burden during tumor progression. The percentage of variant allele frequency (VAF%) for each gene is plotted at diagnosis and at relapse. The names of genes involved are reported under each plot. **c** 7-year PFS in systemic ALCL patients according to *TP53* status; red: *TP53* mutated (mut) patients; blue: *TP53* wild-type (wt) patients. *P* values and hazard ratios (HR) shown were determined by Cox proportional hazards. **d** Schematic representation of p53 domains and the variants detected.

To investigate somatic mutations with a possible role in disease relapse, we sequenced paired diagnostic and relapse samples available for four patients (1 ALK+ and 3 ALK−) (Fig. 2a). Two different acquired mutations in *EPHA5* were detected in each of the two relapse samples (patient tumors ALK+ 1R and HK-10R): a stop codon at S566 and a glycine–valine change at residue 723. In the latter patient (HK-10), identification of mutated *EPHA5* appears to be the result of the emergence of a new malignant clone, harboring novel mutations in several other genes consistently with a similar variant allele frequency (Fig. 2b). Interestingly, *EPHA5* was also found to be the second most mutated gene in relapsed/refractory patients in the entire cohort (Table S4B). Three out of four patients harbored mutated *TP53* both at diagnosis and at relapse (Fig. 2b). As *TP53* is the most recurrent gene mutated in our cohort and the most mutated gene in relapsed patients (Table S4B), we investigated its possible association with the treatment outcome for all ALCL patients regardless of ALK status. Nearly all mutations in *TP53* were detected in the DNA binding domain except for L344P in the TET domain for one patient (Fig. 2d). sALCL patients harboring *TP53* mutations have a shorter PFS compared to those with the wild-type gene (HR = 3.3; 95% CI, 1.59–6.87, *p* < 0.0007) (Fig. 2c). These data, together with the diagnosis versus relapse analysis, suggest that *TP53* mutations may confer resistance to chemotherapy. Moreover, mutations in *TP53* were the most common genetic events on re-analysis of publicly available datasets (Table S7) [9, 11].

Losses at the genomic regions that encompass *TP53* and *PRDM1* genes have been shown to be the most common lesions in sALCL with a clinical implication [12]. *PRDM1* mutations were detected in five patients, with three of these co-occurring with *TP53* mutations and all five patients being categorized within the poor prognostic sub-group. These data confirm the correlation between *TP53* and *PRDM1* gene mutations, thereby demonstrating either copy number loss or concomitant mutations are mechanisms which have the potential to alter p53 and PRDM1 pathways activity.

In summary, within one of the largest cohort of 82 sALCL patients, we provide robust information on the genetic spectrum of genes either solely mutated in ALK− ALCL (*STAT3*, *JAK1*) or across the whole spectrum of ALCL (*TP53*, *LRP1B*, *EPHA5*, *KMT2D*). In addition, we describe novel biomarkers for predicting treatment outcome reporting an association between mutated *STAT3* and *TP53* with an inferior outcome, in the former case in ALK− disease and in the latter case all sALCL independent of ALK status. Finally, this mutational landscape provides further candidate genes that deserve consideration for their possible role in the patient outcome, such as *EPHA5*, *KMT2D*, *PRDM1* and S*OCS1*.

## Supplementary information

Supplementary Information Lobello et al

Supplementary Table S1. Clinical and pathological characteristics of ALCL cohort

Supplementary Table S2. Gene panel

Supplementary Table S3. Variants Identified

Supplementary Table S4 A-C. Main genes mutated in patients with poor prognosis

Supplementary Table S5. Akaike’s informative criteria model

Supplementary Table S6. p-STAT3 immunohistochemistry

Supplementary Table S7. Variants from publish dataset
