# Supplementary material for: STAT3 and TP53 mutations associate with poor prognosis in anaplastic large cell lymphoma
Source: Leukemia. 2020 Nov 27;35(5):1500–5. doi: 10.1038/s41375-020-01093-1 (PMC8102183; doi:10.1038/s41375-020-01093-1)
Supplement: Supplementary file 1 — Supplementary Information Lobello et al [file 41375_2020_1093_MOESM1_ESM.docx]

**Supplementary Information**

***STAT3* and *TP53* Mutations Associate with Poor Prognosis in Anaplastic Large Cell Lymphoma**

Lobello C. *et al.*

**Supplementary Methods**

**Ethical approval**

Formalin-fixed paraffin-embedded (FFPE) tissues, fresh cryopreserved specimens, matched peripheral blood (PB) and related clinical information from sALCL patients were obtained after written informed consent, according to both the Declaration of Helsinki and local guidelines and the ethical approval by the committee from the following institutions: the University Hospital Brno, University Hospital Hradec Kralove (Czech Republic), and the Czech Lymphoma Group Study (CLSG) Registry “NIHIL” (Research ethics committee reference number 4-306/13/1), University of Milano Bicocca (No. 217) (Italy), Medical University Vienna (no. 1221/2019) (Austria) and Children’s Cancer and Leukemia Group (No. 08/H0405/22+5) (UK). Diagnoses were assigned according to the WHO classification of lymphoid neoplasms. DNA from six commercially available cell lines was also used: four ALK+ ALCL (Karpas-299, SU-DHL-1, SR-786, DEL) one ALK- ALCL (FE-PD) and one ALK- cutaneous ALCL (Mac-2A).

**DNA isolation and quality control**

DNA from FFPE tumor sections was isolated using the GeneRead DNA FFPE kit (Qiagen, Hilden, Germany) and the quality assessed using QIAseq DNA QuantiMIZE kit (Qiagen). DNA from cell lines was extracted using Dneasy Blood and Tissue kit (Qiagen), from whole blood samples using MagCore Genomic DNA Whole Blood Kit and from fresh frozen tissue using MagCore Genomic DNA Tissue kit (RBC Bioscience, New Taipei City, Taipei).

**Library preparation, targeted DNA sequencing and variant validation**

Library preparation was performed using QIAseq Targeted DNA – Human Comprehensive Cancer Panel (Qiagen - catalog n. DHS-3501Z) for the enrichment of the coding regions of 275 genes. The quality and molarity of the sequencing libraries was assessed on a fragment analyzer instrument using the High Sensitivity NGS DNA kit (Advanced Analytical Technologies, Ankeny, Iowa, USA) and Qubit HS dsDNA Kit (Invitrogen by Life Technologies, Carlsbad, California, USA). The pooled libraries were then sequenced on the Illumina NextSeq 500 platform using the Mid-Output reagent cartridge 300 cycles (Illumina, San Diego, California, USA). Variants were validated using NGS of PCR amplicons.

**Germinal control**

Sequencing data obtained from DNA isolated from peripheral blood (PB) of sALCL patients has been used as germinal control. Six PB samples from patients from our cohort were sequenced using the same targeted DNA sequencing approach. Fifteen PB samples from patients included in our cohort that were obtained from Gambacorti-Passerini group at the University of Milano (n = 11) (S.G.G. manuscripts in preparation) and the Turner group at University of Cambridge (n = 4)(1) were sequenced using whole exome sequencing (WES). Moreover, we analyzed the data from 23 WES of PB of sALCL from publicly available dataset(2).

**Statistical analysis**

All data analyses were performed in R. *P*-values < 0.05 were considered statistically significant. The Cox proportional hazard model was applied to confirm the relationship between patient survival (overall survival [OS]; progression-free survival [PFS]) and mutational status of genes of interest. OS was calculated from the time of diagnosis to death from any cause or last follow-up. PFS was defined as the time from diagnosis to the first documented event occurring, including relapse, death, or last follow-up. The Akaike information criteria(3) was used as model quality estimator. Survival curves in distinct patient subgroups were computed using Kaplan-Meier analysis. Continuous variables were compared using Mann- Whitney test and categorical variables using Fisher’s exact test.

**Bioinformatics analysis**

Raw fastq files from the sequencing runs were analyzed using two different and independent approaches: Qiagen GeneGlobe software and an in-house bioinformatics pipeline.

In the in-house bioinformatics pipeline raw reads were aligned to the GRCh38/hg38 human reference genome using Burrows-Wheeler Aligner (BWA) tool(4). Aligned reads were grouped by their associated unique molecular identifier (UMI) and a consensus sequence for each UMI group was computed using fgbio toolkit (<http://fulcrumgenomics.github.io/fgbio/>). Corrected consensus sequences were then realigned with BWA. Variant calling was performed with Vardict(5) and Strelka(6) in the single sample target panel mode and resulting variants were annotated with Variant Effect Predictor (VEP)(7). After annotation, single nucleotide variants (SNV) present in any of the above-mentioned germinal controls (n = 44) were filtered out. Variants identified as single nucleotide polymorphisms (SNPs) in databases (dbSNP(8), 1000 Genome Project(9) and MutationTaster(10)) were also filtered out. Two publicly available datasets were downloaded from Sequence Read Achieve (SRA) (identifier SRP044708)(2) and European Genome-phenome Archive (accession number: EGAS00001002740)(11) and the analysis was performed in the similar way only without UMI consensus calling and being the first dataset WES, the variant caller Strelka2(12) was set to a WES mode. Only genes present in our panel (Table S2) were taken in consideration during the analysis of published data. NGS data analysis was also performed using Qiagen GeneGlobe software(13) with the smCounter Version 1.

**Immunohistochemistry**

Immunostaining was performed to assess STAT3 phosphorylation using a p-STAT3 antibody (Y705, Cell signalling Danvers, Massachusetts, USA). Immunohistochemical staining was performed on FFPE-tissue 2,5 μm thick slides, using an automated stainer (Ventana Medical Systems, Oro Valley, Arizona, USA), according to the manufacture’s protocol.

Tumor purity and percentage of phosphorylated-STAT3 (p-STAT3) positivity in the immunostainings were evaluated by two experienced pathologists (F.F. and I.A.M.M.). Percentage of variant allele frequencies (% VAFs) of the STAT3 mutation was correlated with the percentage of p-STAT3 positivity (%p-STAT3).

**Supplementary figures**

**Figure S1**


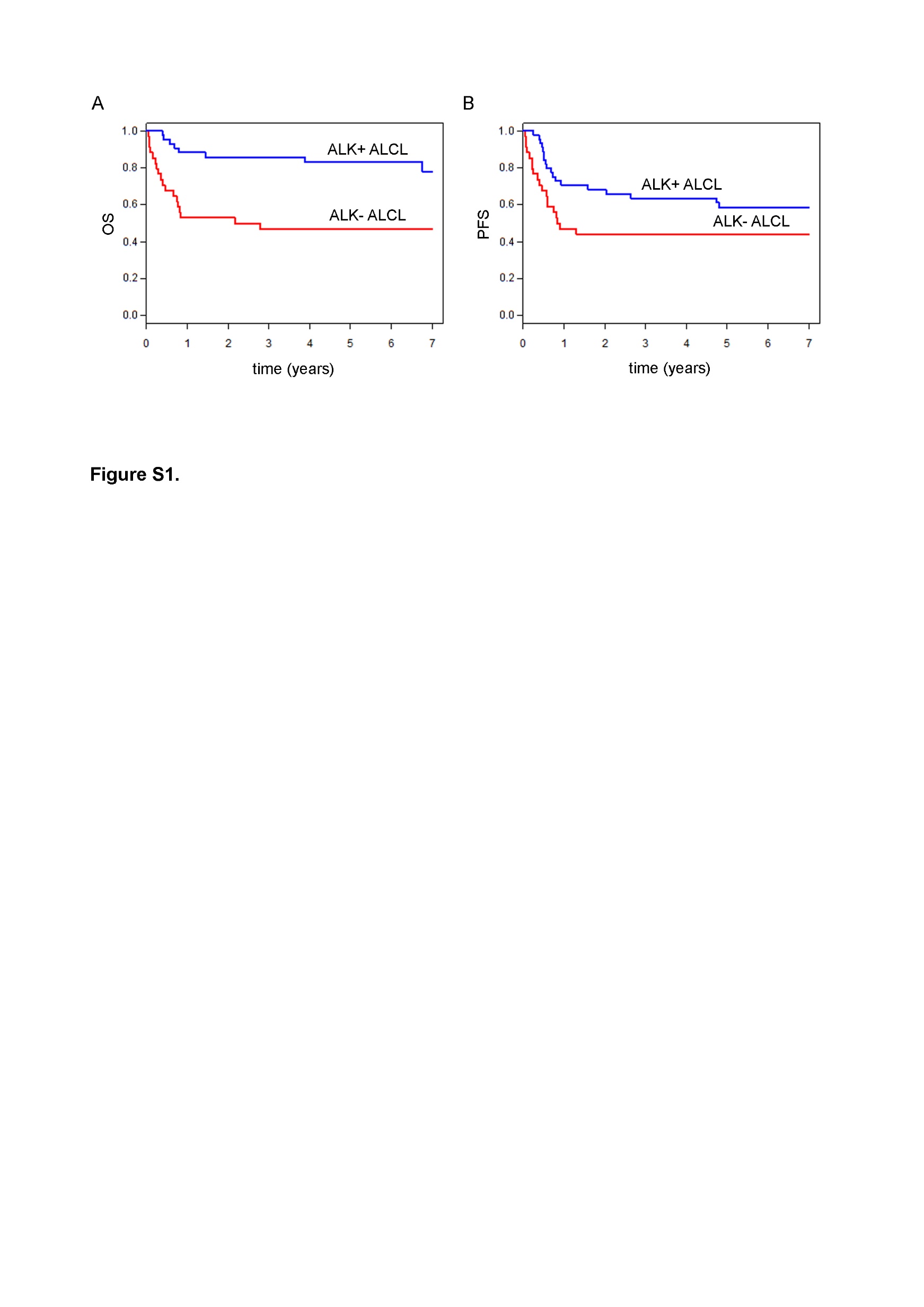


**Figure S1. Overall and Progression Free Survival in the ALCL cohort.** 7-year overall survival (A) and progression free survival (B) of systemic ALCL patients: ALK+ ALCL (blue) and ALK- ALCL (red).

**Figure S2**


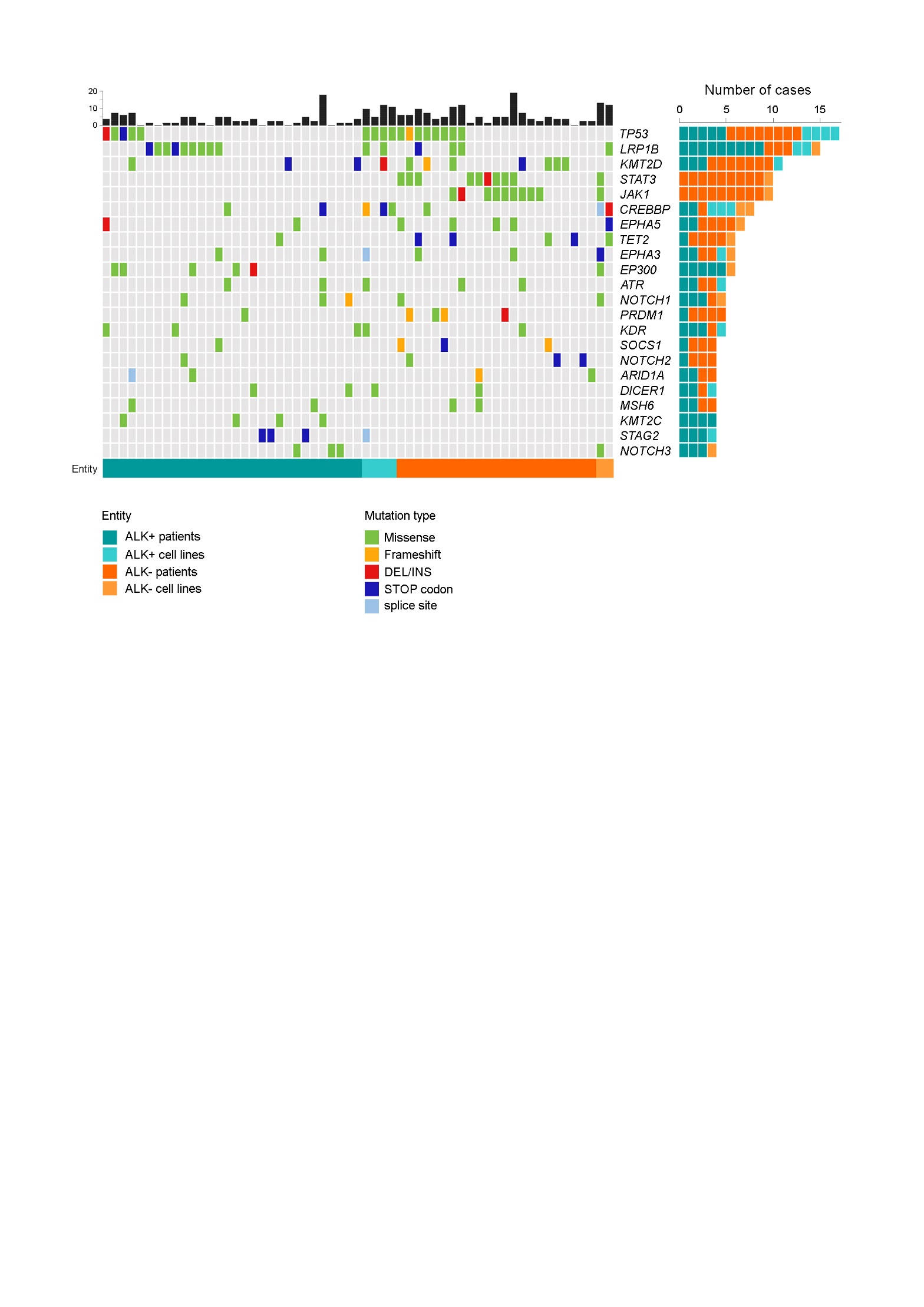


**Figure S2. Mutational landscape in the entire cohort.** (A) Oncoplot shows the most commonly mutated genes found in at least 4 cases, including cell lines. Each column represents a sample, ALK+ ALCL patients (dark green), ALK+ ALCL cell lines (light blue), ALK- ALCL patients (dark orange) and ALK- ALCL cell lines (light orange). The black bars on the top represent the number of mutated genes in each sample. On the right the summary of the number of mutated samples for each gene. Mutation types are represented in different colors as shown in the legend.

**Figure S3**


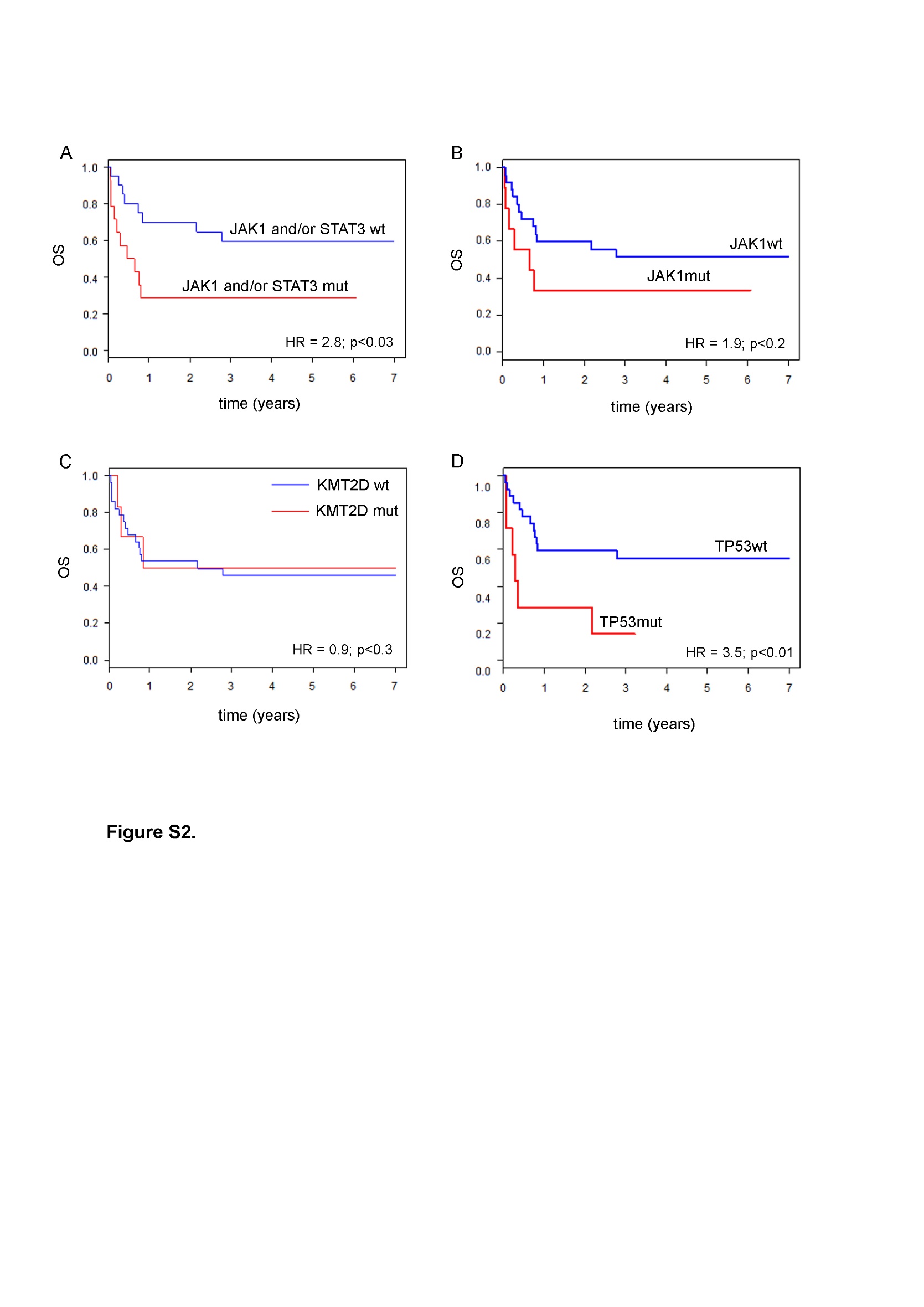


**Figure S3. Investigation of prognostic role of commonest genes mutated in ALK- ALCL**. 7-year overall survival of ALK- ALCL patients according to (A) *JAK1* and/or *STAT3*, (B) *JAK1* (C) *KMT2D* and (D) *TP53* status. Blue: wildtype (wt); Red: mutated (mut). P value and hazard ratio (HR) determined by Cox proportional hazards model are shown.

**Figure S4**


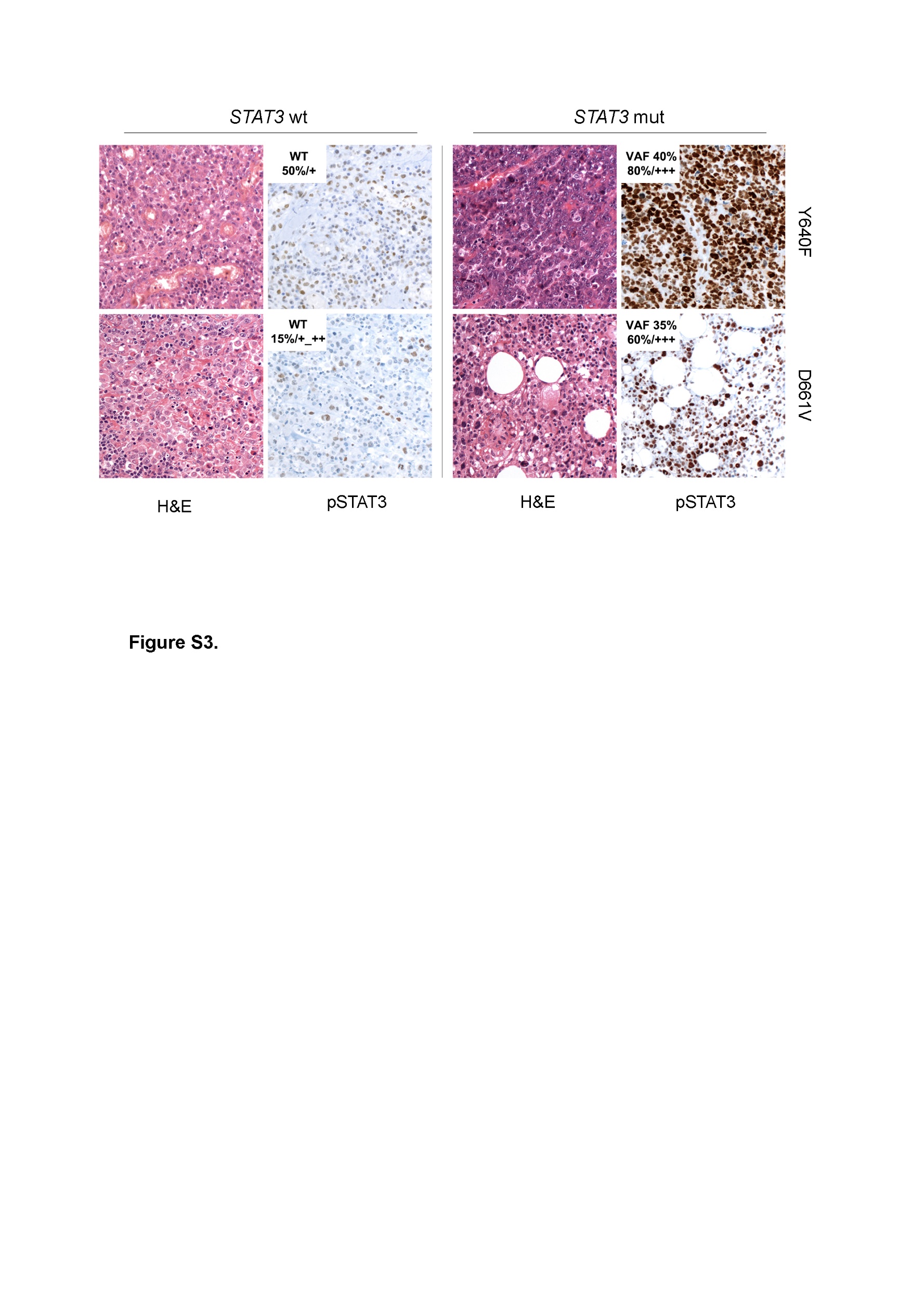


**Figure S4. Expression of phospho-STAT3 in wildtype and mutated *STAT3* patients.**p-STAT3 immunostaining of 4 different ALK- ALCL cases; two harboring wild-type *STAT3* and two mutated *STAT3* (Y640F and D661V). Variant allele frequency (VAF%) of *STAT3* mutation found by sequencing is shown. The percentage refers to the stained tumor cells and the plus to p-STAT3 intensity: + weak, ++ medium, +++ high. Original magnification 400X. H&E: Hematoxylin and eosin stain. In brown stained p-STAT3 for Tyr705.

**Supplementary Tables:**

**Supplementary Table S1.** **Clinical and pathological characteristics of ALCL cohort**. Clinical overview of the cohort. OS, overall survival; PFS, progression free survival; A, alive; R, relapse/refractory; D, dead; ECOG, Eastern Cooperative Oncology Group score; IPI, International Prognostic Index; AA-IPI, Age-Adjusted-IPI.

**Supplementary Table S2. Gene panel.** List of 275 genes sequenced.

**Supplementary Table S3.** **Variants Identified.** List of non-synonymous variants found throughout the whole cohort by targeted DNA Sequencing. * - Stop codon; fs – frameshift, del – deletion; ins – insertion.

**Supplementary Table S4 A-C.** **Main genes mutated in patients with poor prognosis.** A. Mutated genes found in patients with poor prognosis including dead and relapsed/refractory. B. Mutated genes only in patients relapsed/refractory. C. Mutated genes found in ALCL cell lines that were also found in patients with poor prognosis.

**Supplementary Table S5.** **Akaike’s informative criteria (AIC) model**. Results of AIC that show *STAT3* as the best predictor gene.

**Supplementary Table S6. p-STAT3 immunohistochemistry.** VAF% is the variant allele frequency of *STAT3* mutation found by sequencing. The percentage in the IHC: p-STAT3 column refers to the stained tumor cells, and the plus is p-STAT3 intensity: + weak, ++ medium, +++ high.

**Supplementary Table S7.** **Variants from publish dataset**. List of variants found in the re-analysis of published data in genes present in our panel (Crescenzo et al. Cancer Cell 2015, Song et al. Blood 2018).
* - Stop codon; fs – frameshift, del – deletion; ins – insertion.

**Supplementary references**

1. Larose H, Prokoph N, Matthews JD, Schlederer M, Högler S, Alsulami AF, et al. Whole Exome Sequencing reveals NOTCH1 mutations in anaplastic large cell lymphoma and points to Notch both as a key pathway and a potential therapeutic target. Haematologica [Internet]. 2020 Apr 23 [cited 2020 Apr 29];haematol.2019.238766. Available from: http://www.haematologica.org/lookup/doi/10.3324/haematol.2019.238766

2. Crescenzo R, Abate F, Lasorsa E, Tabbo’ F, Gaudiano M, Chiesa N, et al. Convergent mutations and kinase fusions lead to oncogenic STAT3 activation in anaplastic large cell lymphoma. Cancer Cell [Internet]. 2015 Apr 13 [cited 2019 May 17];27(4):516–32. Available from: https://linkinghub.elsevier.com/retrieve/pii/S153561081500094X

3. Akaike H. A new look at the statistical model identification. IEEE Trans Automat Contr [Internet]. 1974 Dec [cited 2019 Aug 5];19(6):716–23. Available from: http://ieeexplore.ieee.org/document/1100705/

4. Li H, Durbin R. Fast and accurate short read alignment with Burrows–Wheeler transform. Bioinformatics [Internet]. 2009;25(14):1754–60. Available from: https://doi.org/10.1093/bioinformatics/btp324

5. Lai Z, Markovets A, Ahdesmaki M, Chapman B, Hofmann O, Mcewen R, et al. VarDict: A novel and versatile variant caller for next-generation sequencing in cancer research. Nucleic Acids Res. 2016 Jun 20;44(11).

6. Saunders CT, Wong WSW, Swamy S, Becq J, Murray LJ, Cheetham RK. Strelka: accurate somatic small-variant calling from sequenced tumor-normal sample pairs. Bioinformatics [Internet]. 2012 Jul 15 [cited 2019 Nov 5];28(14):1811–7. Available from: http://www.ncbi.nlm.nih.gov/pubmed/22581179

7. McLaren W, Gil L, Hunt SE, Riat HS, Ritchie GRS, Thormann A, et al. The Ensembl Variant Effect Predictor. Genome Biol. 2016 Jun 6;17(1).

8. Sherry ST, Ward MH, Kholodov M, Baker J, Phan L, Smigielski EM, et al. dbSNP: the NCBI database of genetic variation. Nucleic Acids Res [Internet]. 2001 Jan 1 [cited 2019 Jun 3];29(1):308–11. Available from: http://www.ncbi.nlm.nih.gov/pubmed/11125122

9. A global reference for human genetic variation. Nature [Internet]. 2015 Oct 30 [cited 2019 Jun 3];526(7571):68–74. Available from: http://www.nature.com/articles/nature15393

10. Schwarz JM, Cooper DN, Schuelke M, Seelow D. MutationTaster2: mutation prediction for the deep-sequencing age. Nat Methods [Internet]. 2014 Apr 28 [cited 2019 Jun 3];11(4):361–2. Available from: http://www.nature.com/articles/nmeth.2890

11. Song TL, Nairismägi ML, Laurensia Y, Lim JQ, Tan J, Li ZM, et al. Oncogenic activation of the STAT3 pathway drives PD-L1 expression in natural killer/T-cell lymphoma. Blood. 2018 Sep 13;132(11):1146–58.

12. Kim S, Scheffler K, Halpern AL, Bekritsky MA, Noh E, Källberg M, et al. Strelka2: fast and accurate calling of germline and somatic variants. Nat Methods. 2018 Aug 1;15(8):591–4.

13. Xu C, Nezami Ranjbar MR, Wu Z, DiCarlo J, Wang Y. Detecting very low allele fraction variants using targeted DNA sequencing and a novel molecular barcode-aware variant caller. BMC Genomics [Internet]. 2017;18(1):1–11. Available from: http://dx.doi.org/10.1186/s12864-016-3425-4
